# Risks of hydroclimatic regime shifts across the western United States

**DOI:** 10.1038/s41598-019-42692-y

**Published:** 2019-04-19

**Authors:** Subhrendu Gangopadhyay, Gregory McCabe, Gregory Pederson, Justin Martin, Jeremy S. Littell

**Affiliations:** 1 Bureau of Reclamation, Denver, Colorado 80225 USA; 2 U.S. Geological Survey, Denver, Colorado 80225 USA; 3 U.S. Geological Survey, Bozeman, Montana 59715 USA; 4 U.S. Geological Survey, Anchorage, Alaska 99508 USA

**Keywords:** Climate sciences, Hydrology

## Abstract

Paleohydrologic reconstructions of water-year streamflow for 105 sites across the western United States (West) were used to compute the likelihood (risk) of regime (wet/dry state) shifts given the length of time in a specific regime and for a specified time in the future. The spatial variability of risks was examined and indicates that regime shift risks are variable across the West. The Pacific-Northwest region is associated with low risks of regime shifts, indicating persistence controlled by prevalent low frequency variability in flow (periods above 64 years). Other areas in the West indicate higher risks compared to the Pacific-Northwest due to flow variability in the mid-to-high frequencies (periods of 32 to 16 years). Understanding risks of regime shifts provides critical information for improved management of water supplies, particularly during periods of extended low flows. The method presented here has global applicability as a decision-making framework for risk-based planning and management.

## Introduction

Water resources planning is a fundamental spatio-temporal risk management problem that is compounded by the combined effects of natural climatic variability and the specific regional expression of the generalized global warming trend. The effects of continued warming on water resources are a concern for societies, industry, agriculture, and ultimately the water managers tasked with allocating this increasingly limited and fundamental resource^1^. In the western United States (West), where the combined climatic demand and consumptive use of water is equal to or greater than the natural supply of water for many locations, there is a general concern regarding the sustainability of future water supplies^2–4^. In addition to the adverse effects of warming on water supply, another issue for water managers is accounting for, and managing, the effects of natural climatic variability, particularly the persistent dry and wet periods that can last years to decades^3,5,6^. Severe sustained droughts and persistent pluvial periods, are a recurring characteristic of hydroclimate in the West^5–8^. Shifts between persistently dry and wet regimes (e.g., decadal-to-multidecadal variability) have important baseline implications for water supply and water management even in the absence of the non-stationary impacts of warming on the amount and seasonal availability of water supply.

Understanding such risks of changes in water supply is ever more critical for an arid to semi-arid region such as the West. The economic consequences of not being able to characterize and use such risk information to make appropriate management decisions are likely substantial. We applied a method to calculate the probability of future decadal-to-multidecadal (D2M) regime shift risks^9^. This methodology has been established to be robust and applicable to any sufficiently longtime series (e.g., multi-century streamflow reconstructions from tree-rings) that exhibits substantial D2M variability^10^.

Across the West, there is a wealth of hydroclimate information contained in reconstructed streamflows derived from tree-ring chronologies, and the primary objective of this research is to use paleohydrologic reconstructions of streamflow to identify and understand the temporal and spatial variability of the baseline risks of regime shifts for sites across the West.

**Figure 1:**
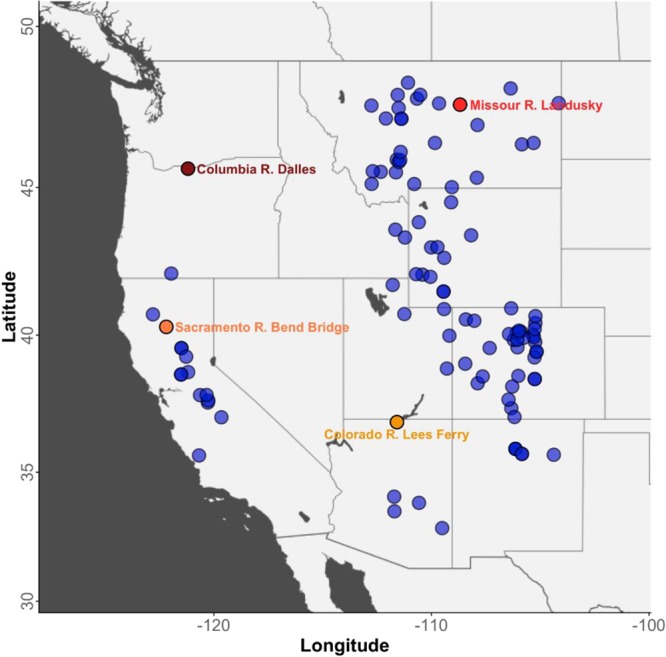
The 105 stream gage locations with tree-ring based reconstructions of water-year streamflow in the western U.S. Four locations used in results and discussions are labeled individually.

We analyze tree-ring reconstructions of water-year flow for 105 sites across the West with complete data for 1685 through 1977 (293 water years; a water year is the period between October 1^st^ of one year and September 30^th^ of the next) (Fig. 1). Figure 1 includes four major river cumulative flow gaging locations that have been labeled as they are specifically referenced in the subsequent analysis. Furthermore, in this study, wet and dry regimes were defined using time series of flow departures from the mean at each of the sites and with variability less than 10 years subsequently removed^11^. The length of regimes, wet (above zero crossing) or dry (below zero crossing) was computed by counting the number of years in each regime for each time series of flow departures.

## Results

**Figure 2:**
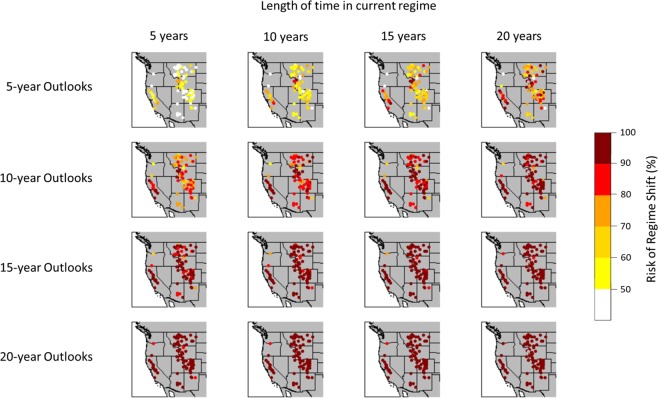
Risk of regime shifts (in percent [%]) for different lengths in a regime and for different periods in the future (outlooks).

The estimated regime shift risk for the 105 sites is shown in Fig. 2. The risk of a regime shift from a wet regime to a dry regime (and vice versa) for each site is illustrated for different lengths of time in a specific regime (i.e. 5, 10, 15, and 20 years) and for different periods of time into the future (outlooks) of 5, 10, 15, and 20 years. We found, the risk of a regime shift is non-uniform across the West, especially for short outlooks (e.g. 5-year outlook). For the 5-year outlook, the risk of a regime shift is relatively low for the Pacific-Northwest (represented by the Columbia River at The Dalles, Oregon gage) and remains relatively unchanged even when the length of time in a specific regime increases to 20 years. In contrast, for most of the other sites, the risk of a regime shift increases as the length of time in a specific regime increases. For example, the risk of a regime shift (in percent) increases substantially for sites in the northern Rocky Mountains when the length of time in a specific regime increases. Additionally, for the Pacific-Northwest, the risk of a regime shift once in a specific regime for 5 years slowly increases from a 5-year outlook to a 20-year outlook, and the risk does not reach 100% even for a 20-year outlook. For most of the other sites across the western U.S., the risk of a regime shift is nearly 100% for the same 20-year outlooks. It is worth noting that the probability of a regime shift over long outlooks would likely be reduced at most of these sites if the length of the common analysis time interval were increased by several centuries. This in effect would allow for the capture and representation of longer dry and wet regimes, and specifically the series of western North American “megadroughts” documented between 800 and 1600 AD^5,6,8^.

**Figure 3:**
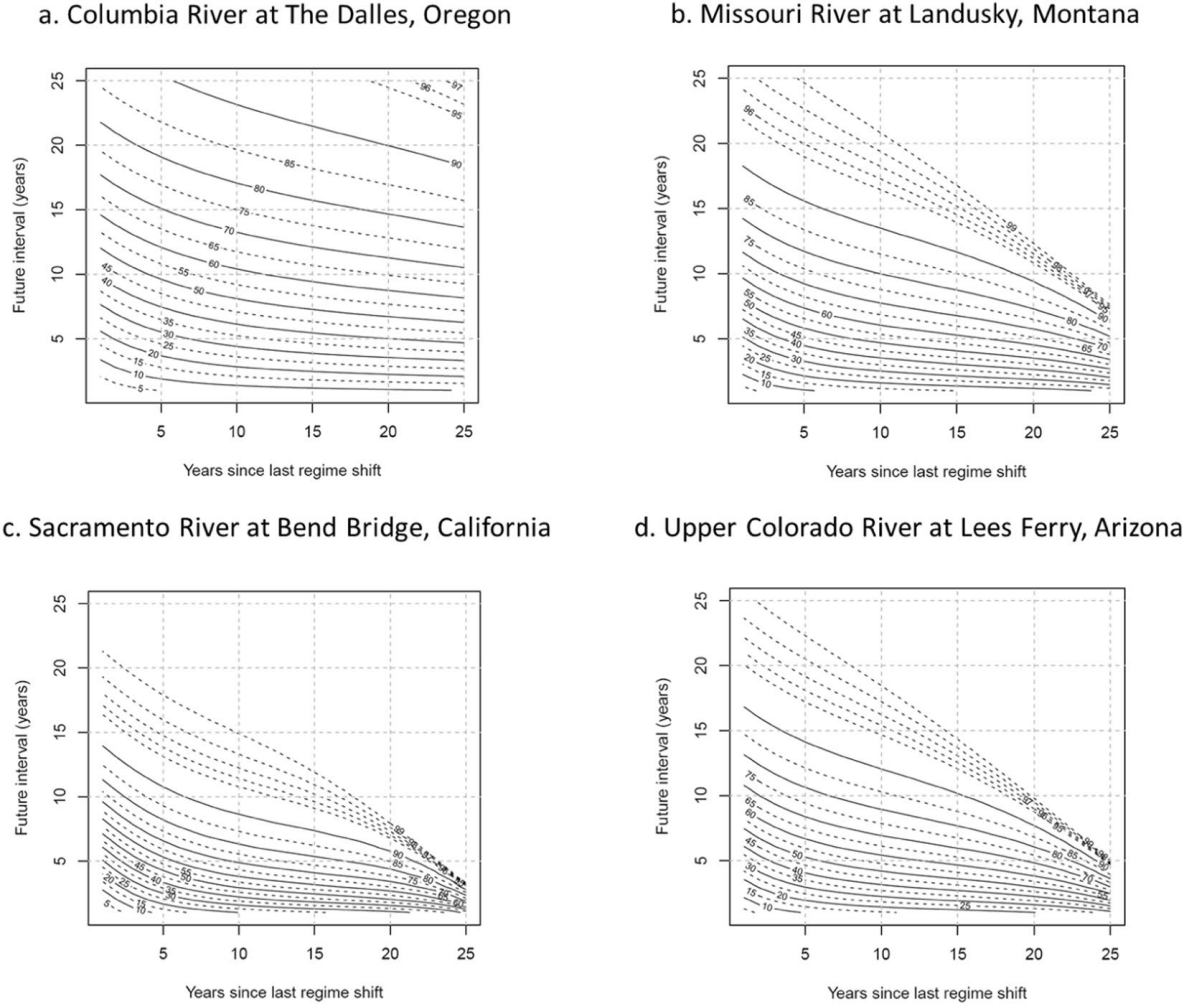
Risk of regime shifts (in percent [%]) for selected basins for different lengths of time in a regime (abscissa, *t*_1_ years) and for different periods in the future (ordinate, *t*_2_ years); truncated for *t*_*1*_ + *t*_*2*_ > 1 year. Note that, a plot like (**d**) has been published previously^10^.

Next, we analyze in detail the regime shift characteristics of the four selected locations that represent major river basin cumulative flow sites. The risk of a regime shift (in percent) is illustrated in Fig. 3 for four sites: (1) the Columbia River at The Dalles, Oregon, (2) the Missouri River at Landusky, Montana, (3) the Sacramento River at Bend Bridge, California, and (4) the Upper Colorado River at Lees Ferry, Arizona. Each of the plots in Fig. 3 shows isolines that indicate the risk of a regime shift for different lengths of time in a specific regime (wet or dry) and for different periods in the future (outlook).

For the Columbia River at The Dalles, the isolines indicating the risk of a regime shift are nearly horizontal to the abscissa and are spaced far apart indicating that the risk of a regime shift for this river does not change much as the length of time in a specific regime increases or as the length of an outlook increases. This result suggests that wet/dry regimes are persistent for the Columbia River basin, which in turn suggests water managers face substantial risks of prolonged high and low flow periods that could be quite challenging for flood and drought operations respectively. In contrast, the isolines indicating the risk of a regime shift for the Sacramento River are closer together and compressed into the lower left-hand side of the plot indicating that the risk of a regime shift for this river changes quickly as the length of the outlook increases. The Missouri River at Landusky and the Upper Colorado River at Lees Ferry both indicate similar patterns of the risk of regime shifts and suggest risks that are generally higher than the regime shift risks for the Columbia River but generally lower than the regime shift risks for the Sacramento River.

To understand the modulation of the regime shift risks, we performed two sets of analyses. First, we analyzed the spectral power distribution for all 105 sites to understand how low frequency variability scales across space and to provide an overall depiction of the regime shift heterogeneity across the West. Second, we analyzed the spectral power distribution at the four selected sites through time for the entire analysis period (1685–1977) to provide context regarding frequencies that drive flow variability and hence the regime shift characteristics.

The spectral power distribution of the reconstructed streamflow across the 105 sites was analyzed using the spectral power-law scaling approach^5,12^. Spatially distributed power-law scaling factor *β* values provide an estimate of frequency variability across space. For a streamflow record that contains significant autocorrelation and increasing variance (or power) with increasing time, a positive estimated spectral *β* value will be generated. The more low-frequency variation present at longer time scales, the larger the positive *β* value becomes reflecting an increasingly “red” spectrum. Near zero spectral *β*. values indicate streamflow records with near equal variance at increasing lengths of time and no autocorrelation (a “white” spectrum); while, negative *β* values indicating negatively autocorrelated streamflow variations with increasing time represents a “blue” spectrum.

**Figure 4:**
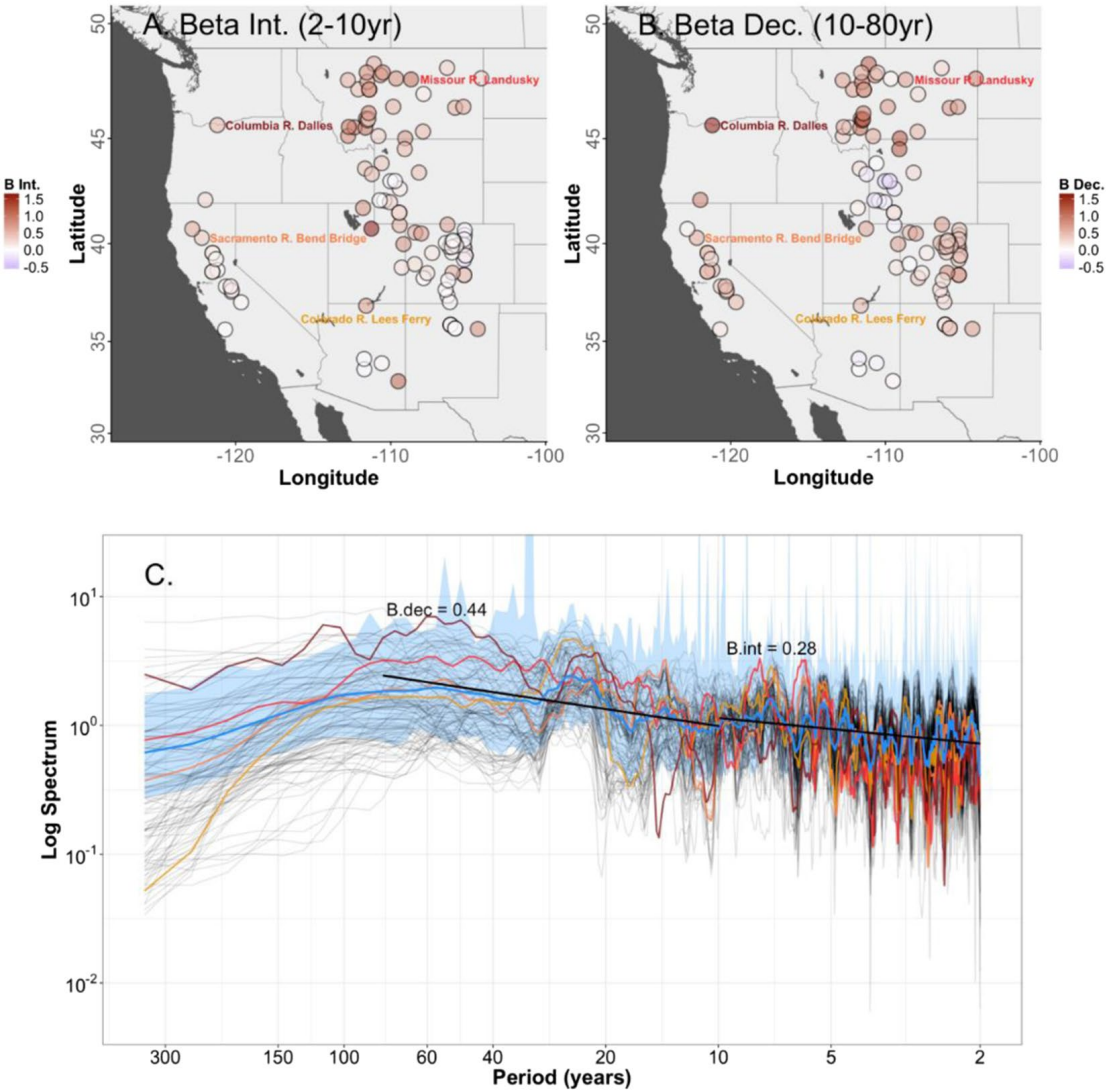
Mapped estimates of (**a**) *β*_int_ and (**b**) *β*_dec_ values for the 105 water-year streamflow reconstructions. (**c**) The individual reconstructions and mean Western U.S. (blue line) spectral density estimates shown with the mean of the bootstrapped 95% confidence intervals (blue shading), and the associated *β*_int_ and *β*_dec_ slopes.

Figure 4 shows the distribution of the power-law scaling factor (*β*) values, which are colored according to the spectral scheme described above (red to blue spectrum), for interannual variability (*β*_int_; 2–10-year, Fig. 4a) and D2M variability (*β*_dec_; 10–80-year, Fig. 4b). The *β*_int_ and *β*_dec_ values have estimated means of 0.28 and 0.44 respectively for the western U.S. (Fig. 4c) and are also spatially variable with generally red shifted spectra at most sites (Fig. 4a,b). Overall, the 2–80-year *β* value estimates range from an insignificant minimum value of −0.18 for a handful of sites in the central Rockies to a maximum of 0.89 for the Pacific-Northwest and a handful of sites in the Missouri River basin. These *β* values generally align with findings from previously published research results^5,12^, showing *β* estimates between 0.2 and 0.8 for precipitation, soil moisture, and the Palmer Drought Severity Index (PDSI) for the western U.S., and *β* values near 0.5 for the globally averaged spectra of river discharge, precipitation, and tree-ring widths.

The spatial variability shown for *β*_int_ and *β*_dec_ values across western streamflow records (Fig. 4a,b) may arise in part due to differences in reconstruction methodological choices, but importantly may also reflect variability that arises from a variety of local to regional sources that are not spatially uniform. For example, the network of 105 sites indicate an increasing spectral power at longer timescales, except for a handful of flow records in the Central Rockies. Also, there is a notable shared peak of spectral power in the 16- to 32-year periodicities (see Fig. 4c) suggesting these are common and dominant modes of streamflow variability across the West and supporting the assumption that all streamflow reconstructions largely retain similar D2M modes of variability regardless of methods used in their construction. The individual power spectra for the four focal cumulative flow gaging locations (color coded to the mapped gage name; see Fig. 1) also highlight the relationship between larger *β*_dec_ values and reduced risks of regime shifts over the range of outlooks (refer to Fig. 4b,c). Overall, the importance of increasing “redness” at D2M scales, combined with spatially variable *β* estimates at interannual through multidecadal scales shown here underpins the fact that risks of regime shifts across the West are most likely not uniform and generally scale according to D2M spectral redness.

**Figure 5:**
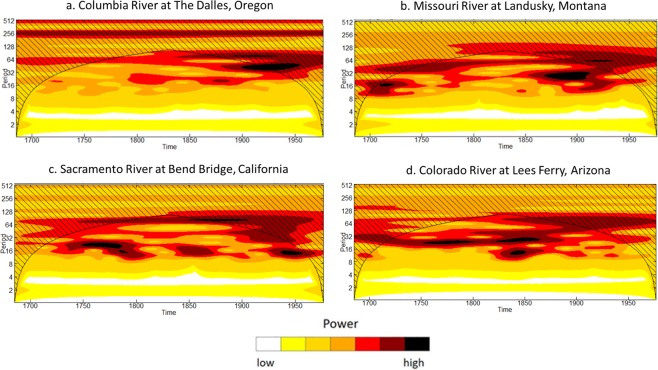
Wavelet analyses of the filtered time series of water-year flow for selected basins. The abscissa shows time (water year) and the ordinate is modulating periodicity (period in year). The cross-hatched regions on either end indicate the “cone of influence,” where edge effects become important^13^. There is minimal to no power indicated for periods below 10 years (frequency, 0.1 cycle per year (cpy)) because of the filtering applied to the time series and the low powers in these frequencies (0.1 cpy and higher; i.e., periods below 10 years) are likely due to the leakage intrinsic in the filtering algorithm.

To further understand the frequency modulation of regime shifts, we performed wavelet analyses^13,14^ of the time series of reconstructed water-year flow for each of the four selected sites (see Fig. 1). The plot of wavelet results (Fig. 5) indicates large amounts of power for variability at low frequencies for the Columbia River reconstructed flow (mostly periods greater than 64 years). The strength of low frequency variability in Columbia River flow is consistent with persistent wet or dry regimes and relatively low risks of regime shifts. In contrast, for the Missouri River, the Sacramento River, and the Upper Colorado River plots of the wavelet results indicate substantial power at relatively higher frequencies (e.g., periods between 16 to 32 years), with the 10- to 16-year spectral peaks notably absent for the Columbia River (refer to Figs 4c and 5). This spectral power at higher frequencies indicates that persistent regimes associated with low frequency variability can be interrupted by relatively higher frequency variability resulting in shorter regimes and greater risks of regime shifts. The wavelet plot for the Sacramento River appears to indicate high power for a larger range of relatively higher frequencies, which explains the greater risks (likelihood) of regime shifts for this river.

## Conclusions

We conclude that the risks of regime shifts vary spatially across the western U.S., especially for short outlooks (e.g. 5 years). The analysis approach using spectral power-law scaling factors in conjunction with site-specific wavelet analysis provides an understanding of the underlying frequency modulation of regime shift characteristics. For example, the Pacific-Northwest is associated with especially low risks of regime shifts indicating substantial persistence of regimes. The large persistence of regimes in the Pacific-Northwest is related to a dominance of low frequency (mostly periods greater than 64 years) variability in flow that corresponds to high spectral power-law scaling *β*_dec_ values, which is generated in part by limited decadal-scale (10–16 year) activity to disrupt this persistence. However, since we used filtered time series with variability less than 10 years removed, we do not account for short-term (or high frequency) climatic variability that can result in a short interruption of a persistent regime such as the one in the Pacific-Northwest. This short-term variability may include La Niña events (with period range typically between 3 to 7 years) which can bring short periods of relief to the Pacific-Northwest when the Pacific-Northwest is in a dry regime. In contrast, El Niño events (opposite of La Niña) can result in a short dry period in the Pacific-Northwest when this region is in a wet regime. Accurate long-lead forecasts require prediction of multi-temporal climatic drivers of streamflow, but in their absence, the tendency (or lack thereof) for regional streamflow regime shifts at specific frequencies allows risk quantification that exceeds random expectations.

Increased understanding of the risks of regime shifts provides water managers with additional information, specifically a risk-based multi-year outlook of likely hydrologic conditions and allows better anticipation of demand and management of water supplies, particularly during periods of extended low flows. For example, Fig. 3d provides an estimate of the likelihood of a regime change in Upper Colorado River flow for specified years in the future. Since the beginning of the 21^st^ century, the Upper Colorado River has been in a mostly dry regime. Since this regime has existed for nearly 20 years, based on Fig. 3d, the risk that there will be a change to a wet regime at 5 years into the future is ~70%, and at 10 years is ~100%. After the Upper Colorado River flow has shifted to a wet regime, the risks of shifting to a dry regime also can be estimated using Fig. 3d.

A topic that requires additional research is how continued warming trends globally will affect the likelihood (risks) of regime shifts. To that end, a study on the Gunnison River basin (a sub-basin of the Upper Colorado River basin) compared regime shifts from paleo-reconstructions of streamflow and projected streamflow from 112 climate model simulations extending through the end of the 21^st^ century^15^. This analysis provided an improved understanding of how the frequency and duration of persistent dry and wet periods may change for the Gunnison River basin as climate changes. Results indicated little change to Gunnison River streamflow regime characteristics through 2039, but between 2040 and 2099, more frequent and persistent dry regimes may increase on the order of 50%. Additionally, wet regimes are expected to be shorter and less frequent than indicated by the paleoclimatic record.

Finally, the available global repository of tree-ring chronologies in the International Tree-Ring Data Bank (ITRDB)^16^ in conjunction with personal and governmental collections could provide an expanded network of available multi-century streamflow reconstructions worldwide. Additionally, re-collection efforts for key tree-ring sites can provide updated and longer reconstructions to recent years. Thus, the robust, data-driven approach presented here has potential global applicability by providing risk-based guidance that can be utilized in planning efforts by the water industry, agricultural operations, economists, and insurers of assets from crops to infrastructure.

## Data and Methods

The streamflow reconstructions and the methods used in analyzing the reconstructions including verification of key assumptions in the reconstructed streamflows for a robust West-wide risk analysis of hydroclimatic regime shift is presented here.

### Tree-ring reconstructions of water-year flow

Tree-ring reconstructions of water-year flow for 105 sites (see Fig. 1) with complete data for 1685 through 1977 (*n* = 293 water years) were used for the analyses presented in this paper. The tree-ring reconstructions of streamflow were obtained from two sources; 74 flow reconstructions were obtained from the web resource, TreeFlow (http://www.treeflow.info/)^17^ and an additional 31 flow reconstructions for the Missouri River basin were obtained from the U.S. Geological Survey in Bozeman, Montana (the Missouri River flow reconstructions can be accessed at 10.5066/P9OYY6DN).

The tree-ring based reconstructions of streamflow represent plausible water-year flow estimates, and no one reconstruction is considered absolutely correct due to a variety of statistical procedures required to convert stand-level, climate sensitive tree-growth variability into estimates of streamflow^17^. Thus, we consider the reconstructions included in this study as reasonable estimates and as representative of the variability in streamflow at each site. However, it should be noted that, streamflow reconstructions can be developed using several different methods and techniques (e.g., biological growth curve detrending choices, pre-whitening chronologies before integration into multivariate regression, and variance rescaling of nested reconstructions) that carry potential implications relative to the preservation of inter-annual to D2M modes of variability. Though within the dendrochronology discipline it is well understood, tree-ring records can capture these modes of variability and individual investigators often seek to utilize methods that reliably preserve these frequencies in the streamflow reconstructions. Thus, it is important to verify that, even with the range of methods independently used by the various investigators in reconstructing streamflow across the West, for the findings presented here to be robust, the reconstructions should be consistent in preserving the temporal modes of variability spatially. This assumption was verified by comparing the power spectra of the reconstructed streamflows for the 105 sites, and the approach is described in the following section.

### Estimating the power spectra of streamflow reconstructions

To test the assumption that D2M variability is preserved, and to assess if generally coherent frequencies of streamflow variability exist across the West, we compare the power spectra of the 105 individual reconstructions using the multi-taper method^18^. Prior to estimating their spectra, each reconstruction was detrended and then normalized to exhibit unit variance over the common 293-year period of record resulting in directly comparable spectral density estimates across records. To understand how low frequency variability scales across space, we build on work from previous researchers^5,12,19^ by estimating and then mapping the power-law scaling parameter (*β*) relating variance to time scale via *S*(*f*) ∝ *f*^*−β*^; where, *S*(*f*) is the power spectrum and *f* is frequency. As in prior work, we estimate the slope (*β*) of the individual power spectrum by estimating the least squares regression of the log-transformed spectral densities against the log-transformed frequencies. We eliminated biasing the regression towards higher frequencies by performing the regression over evenly spaced log-frequency bins for the, *β* interannual (*β*_int_; 2–10-year) and *β* decadal (*β*_dec_; 10–80-year), and overall *β* (*β*; 2–80-year) time intervals. The detrended and normalized time series removed the potential for spectral biases to arise due to the presence of long-term trends and different record means and variance structures. The *β* estimates were made over these specific log-frequency bins to: (1) assess changes in power-law scaling behavior within interannual to D2M frequency windows; (2) limit the *β*_dec_ analyses othe *n* = 293 year long time series matrix to be well within the maximum resolvable period constrained by the Nyquist frequency (*f*_*N*_ = 0.5; with a sampling frequency of one year) of 146 years; and (3) follow prior work in the western U.S.^5^ to generate comparable results. Finally, to represent West-wide spectral power scaling, the weighted mean of the 105 spectral density series was estimated along with the mean bootstrapped 95% confidence Intervals, and mean *β*_int_, *β*_dec_, and the overall *β* values. Interpretation of *β* values is as follows: (1) near zero values describe series with uniform distribution and variance across frequencies with no autocorrelation and are known as “white” spectra; (2) negative values arise in series with negative autocorrelation and are known as “blue” spectra; and (3) series exhibiting increasing variance at longer time scales with significant autocorrelation are known as “red” spectra. Analyses were performed using libraries in the R^20^ statistical computing platform including: (1) multitaper^21^, (2) ggplot2^22^, (3) ggmap^23^, and (4) dplyr^14^. Also, refer to published literature^5,19^ for additional details and examples of estimated *β* values for different hydroclimatic time series.

### Wavelet analysis

Wavelet analysis as used in this study provides an approach to understand the frequency modulation of regime shifts. Wavelets can keep track of time and frequency information^24^ and wavelet transforms can be used to analyze time series that contain nonstationary power at multiple frequencies^25^. Thus, we can identify frequencies (or equivalently periods; *frequency* = 1/*period*) that “dominate” (represented by estimated wavelet power) variability in a hydroclimate series (reconstructed streamflow, here) through time (see Fig. 5). The wavelet analysis in this study was conducted using an approach in published literature^13^ with the Morlet wavelet and an implementation of the approach through a software library^14^ in contemporary statistical computing platform^20^.

### Determining the risks of regime shifts

In this study, a regime is defined using filtered time series of flow departures from the mean at each site with variability less than 10 years removed (i.e. variability at frequencies up to 0.1 cycle per year (cpy) was removed using a Kaylor filter^11^). The high frequency variability in the time series was removed since the focus of this analysis is on the occurrence of persistent dry and wet spells. Although, the time series were filtered (or smoothed), inter-annual variability in streamflow at each site is still represented to some degree in the smoothed time series (e.g., note low wavelet power regions in Fig. 5 for periods less than 10 years are likely due to the leakage intrinsic in the filtering algorithm). This approach also has the effect of limiting any systematic high-frequency bias that could result from comparing flow reconstructions based on different autoregressive methods of estimating mean chronologies. Intervals are defined by periods of successive zero crossings of the filtered time series of flow departures. The length of regimes (wet – above zero crossing or dry – below zero crossing) was computed by counting the number of years in each regime for each flow time series. The reconstructed flow time series provide multi-century records with multiple wet and dry regimes of varying length that are useful to determine statistical distributions of regime lengths.

The assumption here is that the time interval (*T*) between the two flow regime states, dry and wet, is a stochastic process^9^. Given a probability model (*P*) for this stochastic process we can construct useful conditional probability projections for the timings of state change. Details of this methodology, including an application for a single site of reconstructed streamflow, are well established^10^. This study builds on previous work^10^ and expands the analysis to a multi-site approach, providing physical insights through an analysis of frequency modulations and characterizing the spatial variability of regime-shift risks across the West.
